# Past and future corollaries of theories on causes of metabolic syndrome and obesity related co-morbidities part 2: a composite unifying theory review of human-specific co-adaptations to brain energy consumption

**DOI:** 10.1186/2049-3258-72-31

**Published:** 2014-09-01

**Authors:** Anne-Thea McGill

**Affiliations:** 1 School of Population Health and Human Nutrition Unit, Faculty of Medicine and Health Sciences, University of Auckland, Private Bag 92019, Auckland 1142, New Zealand; 2 B-Med Weight Control Consultancy, Auckland, New Zealand

## Abstract

**Forward:**

A composite unifying theory on causes of obesity related-MetS has been formulated and published in an accompanying article (1). In the current article, the historical and recent past, present and future corollaries of this theory are discussed. By presenting this composite theory and corollaries, it is hoped that human evolution and physiology will be viewed and studied from a new vantage point. The politics of management of ecological farming and nutrition will change, a profound reconfiguration of scientific theory generation and advancement in a ‘high-tech’ world can be made, and pathways for solutions recognised.

Metabolic syndrome (MetS) predicts type II diabetes mellitus (TIIDM), cardiovascular disease (CVD) and cancer, and their rates have escalated over the last few decades. Obesity related co-morbidities also overlap the concept of the metabolic syndrome (MetS). However, understanding of the syndrome’s underlying causes may have been misapprehended.

The current paper follows on from a theory review by McGill, A-T in Archives of Public Health, 72: 30. This accompanying paper utilises research on human evolution and new biochemistry to theorise on why MetS and obesity arise and how they affect the population. The basis of this composite unifying theory is that the proportionately large, energy-demanding human brain may have driven co-adaptive mechanisms to provide, or conserve, energy for the brain. A ‘dual system’ is proposed. 1) The enlarged, complex cortico-limbic-striatal system increases dietary energy by developing strong neural self-reward/motivation pathways for the acquisition of energy dense food, and (2) the nuclear factor-erythroid 2-related factor 2 (NRF2) cellular protection system amplifies antioxidant, antitoxicant and repair activity by employing plant chemicals. In humans who consume a nutritious diet, the NRF2 system has become highly energy efficient. Other relevant human-specific co-adaptations are explored.

In order to ‘test’ this composite unifying theory it is important to show that the hypothesis and sub-theories pertain throughout the whole of human evolution and history up till the current era. Corollaries of the composite unifying theory of MetS are examined with respect to past under-nutrition and malnutrition since agriculture began 10,000 years ago. The effects of man-made pollutants on degenerative change are examined. Projections are then made from current to future patterns on the state of ‘insufficient micronutrient and/or unbalanced high energy malnutrition with central obesity and metabolic dysregulation’ or ‘malnubesity’.

Forecasts on human health are made on positive, proactive strategies using the composite unifying theory, and are extended to the wider human ecology of food production. A comparison is made with the outlook for humans if current assumptions and the status quo on causes and treatments are maintained. Areas of further research are outlined. A table of suggestions for possible public health action is included.

## Background

The degenerative disorders associated with major health problems such type II diabetes mellitus (TIIDM), atherosclerotic cardiovascular disease (CVD) and cancer have increased markedly over the last 100 years or so. Almost all populations suffer from high rates of these diseases in the 21^st^ Century. In the last five decades obesity rates have accelerated and are associated with the above degenerative conditions. They are linked, and predicted by, a cluster of markers comprising hypertension, dyslipidaemia and hyperglycaemia, denoted the metabolic syndrome (MetS).

A change to an individualistic, capitalist, westernised way of life has contributed. High energy, refined, over-appetising food has always been a profitable commodity. Science prioritises high-technology oriented commodities that can be mass produced for high financial returns. However, processed food has little overall nutritional value, and its production also involves significant amounts of anti-nutrient additives. Such high energy refined food is known to influence obesity, but the mechanisms of its contribution to MetS are not understood. Much public health and ecological science has been developed to try to rectify the issues caused by technological production of food.

However, some cultures have been pursuing this pattern of increasingly designing and using new technologies since agriculture and animal husbandry arose. The foraging or a hunter/gatherer way of life was effectively that in which humans evolved and for which they were most adapted. Even primitive farming meant a significant change in nutrition, which may need accounting for.

As effective methods to deal with human obesity, let alone basic causes of MetS, are unknown, a review of human evolution and history is required in order to form a unifying theory. Any such theory is likely to be a composite of various hypothesises and extant or new sub theories. Once such a composite unifying theory is formulated it needs to be ‘tested’ against what we know of all of the changes humans have made or have occurred to them, since they left their time of maximum fitness and health.

The composite unifying theory has been developed [1]. It states that human physiology, in addition to anatomy, changed during the evolution of the proportionately large, energy-demanding human brain. Such co-adaptive mechanisms were required to provide or conserve more energy for the brain. There are a number of human specific mechanisms that may be contributing to human obesity. However, two co-dependent, but unrelated co-adaptive systems or a ‘dual system’ is proposed: 1) the enlarged, complex cortico-limbic-striatal system increases dietary energy by developing strong neural self-reward/motivation pathways for the acquisition of energy dense food, and 2) the nuclear factor-erythroid 2-related factor 2 (NRF2) cellular protection system amplifies antioxidant, antitoxicant and repair activity by employing plant chemicals, becoming highly energy efficient in humans.

These two systems may have come to interact in a negative way since technologies advanced.

The complex human cortico-limbic-striatal system generates strong behavioural patterns, based on neural rewards, for energy dense food procurement, including motivating the development of agricultural technologies and social systems to augment the process. Once significant amounts of this high energy, refined food are produced for communities, the cortico-limbic-striatal reward system tends to an extreme, ‘overdrive’ setting or addiction in many individuals. Aspects of addiction include lack of control of the behaviour and over-consumption of the salient item, and neglect of healthy behaviours such as eating nutritious but less appetising ‘common or garden’ food.

Insufficient consumption of food micronutrients, such as secondary plant food micronutrients, prevents optimal human NRF2 function. In humans, the micronutrient dependant, hyper-functioning NRF2 appears to protect long lived cells, such as cardiomyocytes and neurones, from oxidative stress, thus conferring many decades of excess functional longevity. Additionally, the human NRF2 protects fast turnover immune/endocrine or other epithelial cells from toxins, stabilises and controls replication, which decreases rates of neoplastic change and cancer. If there are insufficient food micronutrients, inefficient oxidation of excess energy forces central and non-adipose cells to store excess toxic lipid. Lack of detoxification of foreign or unnecessary (xenobiotic) chemicals exposes cells to toxins which poison many cell processes, introducing dysfunction or apoptosis. Ensuing oxidative stress and metabolic inflammation (metaflammation) allows susceptibility to infectious, degenerative atherosclerotic cardiovascular, autoimmune, neurodegenerative and dysplastic diseases.

### Corollaries of the composite unifying theory on the metabolic syndrome

This composite unifying theory has a number of corollaries which modify current ideas on why human health has changed over the ages since agriculture began, what is occurring now, and what the future could bring.

### Nutrition through the ages - agriculture, industrialisation and migration

The health effect of the cortico-limbic-striatal system’s driving humans to attain a high energy diet can be traced over the last few millennia. As food processing technologies developed, low micronutrient to macro-nutrient ratio diets became more frequent rendering the high micronutrient dependant energy efficient antioxidant NRF2 system increasingly dysfunctional.

The archaeology of pre-agricultural era humans confirms that they were taller, more muscular and robust [2]. Although the average life span was only 40 years, it seems to have been kept lower by high infant mortality. Healthy old age did occur [3].

The advent of farming technologies dates from about 10,000y ago [2]. Energy dense, storable and transportable, starchy foods increased to 50-70% of the diet and the diminishment of variety was significant [4]. Such diets allowed rather more predictable, year round energy supplies, and humans to breed, migrate and colonise much of the world. However, there was a health cost to the loss of a widely varied, whole food diet [2]. There is evidence that many, but not all, agricultural populations became stunted; even brain size decreased [3].

The mean height of pre-19th Century European women was approximately 155 cm, and of men, 166 cm, with men losing more highly nutritionally-demanding, lean tissue [5]. European mediaeval city dwellers became more disease, particularly infection, prone,[2] with average life expectancy of 18 years [3].

The lack of vitamin D, for instance predisposed humans to the chronic infections of tuberculosis, leprosy and syphilis [6], in addition to rickets, but has probably had unrecognised nutrition-related metabolic influence in the liver [7]. It is worth noting, at this point, that neoplastic tumours were rare in non-industrialised communities [8]. In addition, the development of the Northern Hemisphere human’s minimally pigmented skin was probably an adaptation to lack of skin sun exposure for synthesis of vitamin D [9]. Infection and bone dysplasia probably also exerted epigenetic, if not genetic, selection [10].

Lack of micronutrients was likely to have profound effects on human health, and recently realised now that analytical techniques have advanced, is that of dietary quality (energy and micronutrient type, variety and volume) on intestinal microbiota [11]. A few studies have associated not only the energy content, and energy harvest, of diets with more or less beneficial microbiota dominance, but also the type of food culture, ethnicity and degree of westernisation [12].

Interestingly, there are a number of other genetic adaptations which may have occurred in response to humans occupying self-made niches, and cultural practices [13].

Early in the 20^th^ century, an increasing understanding of microbiology led to improved public sanitation and nutrition in some westernised populations. Later in the century, antibiotics were developed and significantly decreased puerperal and infant infection, contributing to increased maternal and infant survival [14], and increased average life span. In addition, the discovery of the requirement for most vitamins and minerals reassured consumers that they just needed a few supplements for health. The rapid escalation and success of supplement supplier and advertising industries followed and continues [15]. Processed, highly palatable fortified foodstuffs, along with synthetic supplements, including infant formulae, became preferable to previous low processed diets. The convenience and sophisticated advertising of refined energy foods are strong cues, and cues feed into, or are a part of, addictive behaviours [16]. Finally well entrenched habits form [17].

### Nutrition and lifestyle, and medical and surgical, treatments for obesity and metabolic syndrome

Early, and recent, TIIDM and CVD public health research and ‘lifestyle’ messages focussed on behaviours related to decreasing dietary energy and increasing physical activity [18]. However, various low energy, set-item diets, often commercially formulated as high dairy protein, vitamin and mineral fortified meal replacements, only work well in the obese if they are well below the average adult daily energy requirements, and then with much health professional support. ‘Calorie counting’ by individuals in the community on ad libitum diets usually fails in the long term, which is possibly due to the retention of addictive, refined energy foods, and insufficient fruit and vegetable intake.

In those with MetS who do make the change to a whole food type of diet, or even a 'higher fruit and vegetable and lower salt' diet such as the Dietary Approaches to Stop Hypertension (DASH [19]), weight loss and improvement in many metabolic biomarkers can be shown [20]. Further, the long running Mediterranean intervention PREDIMED study (including groups where olive oil and nuts were provided), was recently stopped early as CVD events and death rates were unacceptably high in the low-fat control group participants [21].

In almost all clinical studies, humans who habitually consume *diets,* such as from the traditional Mediterranean region, tend to maintain normal weight with decreased risk of CVD and cancer [22]. These type of traditional whole food diets are higher in low-processed, synthetic-chemical ‘free’ , hardy, old varieties of fruit and vegetables, which make their own secondary or defence chemicals. Such diets include mixed protein from plant and free range animal sources. These diets do not have to be low energy, and notably, the Mediterranean diet is proportionately high in energy from cold pressed, high polyphenol-content oil, in addition to fats in dairy, meat, fish, and nuts. The typical traditional Mediterranean contains approximately 38% of olive oil, equivalent to the fat percentage of a typical western diet [23]. Note that saturated fat has been present in our diets for millions of years [24]. It appears high ‘fat to carbohydrate’ ratio, and even ketogenic, diets are physiologically not a problem generally or in obesity [25].

Many researchers have concluded that a narrow range of olive oil and wine polyphenolic metabolic activity was the main or only mechanism for the Mediterranean diet’s health properties [26], rather than the total nutrient content and proportion. This belief possibly spurred on the super-food and nutraceutical industry in its search for one or a few commercialisable food extracts to marketeer. With each new piece of research published, individuals are buffeted back and forth on how to make nutrition choices [27]. Humans have minimal awareness of micronutrient deficits, although they may overeat refined energy food as they seek protein [28].

Epidemiological studies show dietary intakes of whole foods, including their antioxidants, are associated with better health [29]. However, there is accruing evidence that mono or multi-vitamins have neutral-to-negative effects [30] including mineral supplements [31], when given therapeutically. Antioxidant supplements can perturb important, controlled, ‘positive pro-oxidant effects’ , including mitohormesis (cellular regeneration due to small negative stimuli) in post-exercise muscle [32,33], infection response or inflammation and, recently realised, cancer prevention [30]. In spite of the lack of evidence of efficacy [34], a multibillion dollar a year supplement industry flourishes for humans and animals [15].

Modestly increasing fruit and vegetables in the diet has gained some traction and become a widespread health message. However, it coexists with muddled information from researchers, clinicians and public health professionals, let alone the refined energy food industry, on how much grain and tuber starch, fruit sugars, hydrogenated oils and animal fat should be consumed.

In addition, a vast pharmaceutical and medical device manufacturing industry flourishes for the treatment of blood pressure, lipid and carbohydrate markers of MetS [35], which further distracts from problems with human metabolism and nutrition in the current environment.

Bariatric surgery should be mentioned as it is often held up to be the ‘gold standard’ of weight loss. It is available to relatively few [36] and can result in massive weight loss, although early and late surgical problems do occur and can require revision. However, the post-surgical diminished volume/energy diets are rarely high in whole food micronutrients. Patients are prescribed unproven multi-vitamins supplements, although this practice, and compliance, is highly variable [37]. Total nutrient malabsorption and micronutrient deficiencies can be the cause, or result, of metabolic derangements [37]. Decreased morbidity and mortality is not assured in bariatric patients with established MetS [38].

Long term follow-up in many reports is patchy, and weight regain is not rare [36]. In the short term, as bariatric patients recover from variably extensive surgery, cell triglyceride mobilisation and ‘downsizing’ cell organelle components (molecules and minerals) are recycled (autophagy). The released cell molecules increase micronutrient to macronutrient ratios. This may be the reason for the (often temporary) improved insulin sensitivity and general metabolism, including regression of TIIDM, rather than the proposed “lower intestinal hypothesis”. This hypothesis is ‘based on the documented substantial changes in incretin and other entero-endocrine responses’ [36]. Note that incretin treatment may increase pancreatic hyperplasia and sequelae [39].

However, for bariatric patients who do make significant changes to appropriately-prepared whole food diets, and who develop regular physical activity patterns, the regain of health can be gratifyingly long-term, and significant.

The above discussions revolve around 1) modestly low food micronutrient to macronutrient ratios with deficient overall food volumes contributing to stunting in the past few hundreds to thousands of years, and 2) the extremely low food micro to macronutrient ratios with overall excess energy food associating with central obesity pandemic in the last few decades to the present. However, between these two ‘eras’ , another type of degenerative problem appeared.

### Toxicity as a cause of metabolic syndrome – cardiovascular disease and cancer

From the early 1920’s to the 1960’s, as infectious disease rates plummeted, another significant disease markedly increased. MetS, without (obvious) central obesity and specifically atherosclerotic ischaemic heart disease (IHD), rates became epidemic. Lifestyle change data has not explained all of the increase in CVD. An alternative explanation is possible for IHD which escalated in incidence in studies of lower middle to middle-class men from westernised countries during the late 19^th^ and early-mid 20^th^ century [40]. Over this period widespread war and work with vastly increased heavy industrial machines and materials were associated with increased smoking rates, stress, poor sanitation, sub-standard nutrition and crowding.

Importantly, and probably under-recognised, was the general and specific exposure to new synthetic persistent chemicals. All of the above factors probably contributed to traumatic, toxic and infective death rates, particularly in men. Is posited here that MetS and sequelae may be a partial response to variably toxic man-made pollutants which were released with few controls, and importantly coupled with diets insufficient in micronutrients [41]. It is likely that the problem started in towns of the industrial revolution, and later moved into industrialised agriculture and warfare.

The most well-known association of toxins and CVD and cancer is, of course, with chemicals from burnt plant derivatives: coal, oil and tobacco. A famously recognised early example is scrotal cancer in chimney sweeps [42]. Tobacco smoking has persistently and unequivocally been shown to associate with all degenerative disease [43]. The peak CVD rates in men occurred after the second world war [44], with significant declines in this same group of men not starting until the late 1960’s [45]. Interestingly, smoking was proven to cause both CVD and cancer in the same period. The United States of America (USA) Surgeon General [46] announced this finding and that smoking cessation was to be encouraged. Concurrently, awareness of the need to control the environmental release of acute and persistent pollutants was popularised by publications such R Carson’s ‘Silent Spring’ [47]. Increased numbers of, and more effective, pollution control measures were instigated thereafter [48]. Environmental toxins, including smoking, may explain why nearly half of the decline in CVD cannot be attributed to low fat and sugar diets, new MetS pharmaceuticals and CVD interventions [49,50].

The association of industrial chemicals on associated acute illness [51] and cancer development [52] has been investigated. However, most research on cancer has been concentrated on non-industrial exposures such as domestic fires and aflatoxin [53], especially in less developed countries. There is little emphasis on long term persistent chemical exposure and CVD in the general medical literature, even now [54,55].

Currently, a high proportion of the world’s communities are exposed to modest to significant doses of thousands of types of persistent (organic) pollutants and other xenobiotics [54-56] and the lowest micronutrient to macronutrient ratio in human evolution. Scientists are often under political pressure to commercialise research to produce various technologies that support industry, and food science is a very active area [57,58]. Chemical and mechanical technologies are constantly being developed for food: preservation, travel, shelf-life, acceptable look and size, energy content and palatability, rather than nutrition. Nutrition science is perhaps a poor cousin of food science, and toxicology is often not prioritised [59]. Whilst there is a long history of clinicians who profit from industry involvement [60], it is likely that most public health nutrition scientists and physicians believe that they are trying to advance science in good faith. Public health researchers are often under considerable pressure from governments to not upset industry. However, there are a few public health units and directors who have managed to maintain hard hitting, unbiased research that reveals the extent of the influence of industry on nutrition [61,62]. Data on the effects of sugary drinks and their contribution to obesity and poor health are conclusive, as are the marketing tactics of Big Food [61].

### Malnubesity

The current cohort of humans has been unable to efficiently oxidase their excess energy. This leads to ectopic, toxic lipid accumulation [63] in perivisceral, upper body and organ tissue, as seen with central obesity. This state of excess oxidative stress and ineffectively detoxified xenobiotics [64] in the suboptimal, micronutrient-deprived NRF2 system leads to the inability of cells to perform maintenance and autophagic repair work [65]. Fast-turnover cell replication loses its strict regulation and tends to dysplasia, with immune cells particularly being prone to acquired DNA damage; dysfunction or malignancy rates increase [30].

Metabolic inflammation (metaflammation [66]) occurs, especially in the arterial endothelium (as atheroma containing oxidised, pro-thrombotic, lipid engulfed by cytokine-secreting foam-cells), endocrine epithelium (dysplasia), liver (production/secretion of inflammatory proteins & dysplasia), cardiomyocytes (ischaemia/lipotoxicity [63])) and central nervous system protein damage by glycation and misfolded protein (neurodegeneration [65,67]). Many other related mechanisms occur, often overlapping in different tissues. This could be called malnutritive obesity or malnubesity [68].

#### ***Future scenarios***

As can be seen, many researchers and practitioners have undertaken work in multiple fields that relate to the problem of MetS. Some have known that there are many unsolved issues relating to human evolution and development. Evolutionary studies and basic science, especially cancer bioscience and addiction research on the NRF2 and cortico-limbic-striatal systems respectively, have unwittingly provided plenty of evidence for the ‘dual system’ idea. These, and many other disciplines contributing information on, for example, the human propensity to gain fat and develop slowly, allowed for the pulling together of the many parts of the composite unifying theory.

There is still research to do in rigorously studying the effects of an *ad libitum* whole food diet in humans under controlled conditions on MetS and other markers. Mathematical modelling and energy balance studies in obese humans on whole food diets are required to deduce how much of the whole food diet contributes to the NRF2 energy efficiency effect and how much is a vegetable fibreenergy dilution effect. Field trialling is required to study various models of ecologically-sustainable, healthy, mixed crop and stock farming using traditional and historic, as well as current smart technologies and techniques (Table 1). This type of farming should produce decreased volumes of lower energy/higher nutrient-value food [69].

**Table 1:** Public health responses to the composite unifying theory for decreasing metabolic syndrome & obesity related co-morbidities

**Type & putative reason**	**Mechanisms**	**Effects on human form & function subsequent relationship to disease**	**Public Health Policy & Action**
Expanded Cortico-Limbic-Striatal System (human specific/ adapted) [Increased Energy Uptake].	• The cortico-limbic-striatal system is present in mammals (palaeo-mammalian brain derived from reptilian elements). The system ensures behaviours follow basic drives for food energy, sex, power etc. It controls planning & motor co-ordination, with motivation enhanced by significant neural reward in humans	• Sociological literature has dealt with the huge disparities in power between individuals in populations, usually to acquire or manage resources such as food production & human labour. This is typical of human society, however ‘power addiction’ as it relates to public health, is rarely discussed.	• Public health is about promoting & ensuring healthy environments & the rights of all to health. Basics, such as nutritional & safe food, remain pivotal. In practice, often indirectly, it involves defending those with limited resources for healthy lives from those who have (expropriated) more resources. Governments, which largely fund public health programmes & should have the same aims as public health, are highly influenced by profitable industrial lobbies. Lobbyists & politicians’ jobs are all about power, so any ‘addiction to power’ is an issue for public health. Ethical considerations & scientific evidence may not always shape laws which politicians devise & pass. Big Food, a typical profiteering industry, has a powerful, multinational lobby; whole food producers are the converse.
	• Reward is determined by dopamine function, & mediated by many endogenous & exogenous neurotransmitters such as opiates, cannabinoids etc. by direct stimulation or by items/situations of learned salience (initially pleasure)	• Highly driven/perseveration behaviours with feed -forward reward systems may have transferred to many pursuits, including discovery, invention & technology (often designed to increase energy acquisition), gaming, gambling, & systems of power over others	• Public health workers have an number of options, which could include to a) become educated in tertiary institutions with a high degree of academic freedom b) work out an ethical code with(in) which to work c) critique all research & studies for adherence to sound hypotheses & methods d) have a working knowledge of new basic science developments, changes in macroeconomic trends & effects of technology e) collaborate with & harness the support of the many civil society groups
	• In humans, there is a large expansion of this system in form & function – with the dopamine transporter showing recent rapid evolution, high degrees of variation in genetic variability & epigenetic responses.	• ‘Reward hypo-function’ requires more of the ‘stimulant’ in order to function; enjoyment is often no longer present	• Public health system staff & policy makers will face resistance from themselves personally – a) why should they personally cut down on refined & appetising foods, b) why should they resist a main source of income as much research is carefully backed by food processors/marketeers, c) why should they fight their colleagues in nutritional, supplement, pharmaceutical, food & agribusiness all for a theory.
	• Increases in &/or repeat seeking behaviours can be extreme (perseveration, obsessive- compulsion)	• Obsessive compulsive disorders commonly follow, & highly cued, addiction ‘habits’ develop.	• Public health can support dealing with addiction as biology, not buying into blame of sufferers with statements on ‘individual responsibility’ – which certainly does not help children
	• Addiction can occur or restart in ‘permissive’ environments, or when individuals are under stress	• ‘Refined food addiction’, loss of control, binge eating over-palatable food, distress, guilt, depression, is so strong that obesity commonly supervenes, in spite of marked social disapproval & derision	• Public health workers on the ground & primary care health professionals, need support for early addiction detection & help with addiction therapies:- non-judgemental counselling, abstinence from certain foods, replacing the refined high energy food addiction with healthy foods & habits, & use of safe anti-addiction medication
	• Addiction relies on cues, & habit formation: can be extreme (obsessive-compulsive perseveration)	• Obsessive compulsive disorders - includes anorexia nervosa, bulimia & many others	• Public health messages are frequently low impact, non-productive, admonishment of families’ behaviour & composed of soft, biologically outdated lifestyle advice. Public health workers could work to understand how not to buy into manipulative messages in the guise of ‘social marketing’. These are part of the tactics of Big Industry, to influence ‘media messages’ & normalise addictive items to populations. Such messages reassure people that their behaviour does not need much change; certainly not stopping. Messages such as ‘just having these foods as part of a balanced diet’ or, with respect to alcohol, ‘cut down on drinking’ are counterproductive for people with addiction.
	• Addiction to power is little discussed in these terms, but probably stems from in same part of brain	• Addiction arises when power & technology systems make salient items easy to attain, & promise rewards of feeling better	• Public Health workers need to be aware of being steered into working with industry or having industry provide funds, especially to study industry- beneficial or deleterious issues. Governments tend to prefer to fund public health research on neutral epidemiology of illness rather than psychosocial issues associated with societal disparity. Public funding is rarely enough. Better designed, prospective preventative, positive role modelling community studies are needed.
		• Loss of control over items/situation, leads to neglect of healthy & normal behaviours, such as healthy eating	• Public health could support & work with civil society groups in a mutually beneficial way, & possibly consider ‘crowd funding’ to get projects going.
		• The dopaminergic system (as opposed to the serotonergic) is not ‘linked’ to logical/cognitive part of brain; talking individuals out of addiction feelings is not successful. Non-judgemental help to understand what addictions are, how rules around behaviours have to be made & ‘policing’ them, all need external input & support.	• Public Health staff need to keep trying to ensure advocacy & regulation to prevent public health damage from addiction & exploitation. Regulating where alcohol is sold & what food is available in schools, are derived strategies learned from tobacco control.
			• Public Health staff should work to prevent Big Food/Alcohol/ Tobacco, Energy, transport industries lobbying with gifts to regulators. They should ensure totally transparent dealings with governments, & cessation of large direct & indirect public subsidies made to such industries.
Omnivory (human adapted) [Increased Energy Uptake].	• Omnivory increases energy by exploiting high energy/protein/nutrient animal tissue –lean (mostly muscle), fat (plus marrow, brain) offal (liver, kidney, & other). Omnivory continues use of (seasonal) ripe fruit with sugars, oil seeds (note high-starch foods were rare in pre-agricultural times).	• Wide varieties of plant & animal food were consumed & experienced by early humans, & gradually became required nutrients.	• Farming should be mixed, with a much higher ratio of plant protein/micronutrient rich food to plant starches, sugars & oils (the latter grown for industrial refinement).
			• More animals & fish can be grown on mixed farms using organic waste such as carp in farm sludge ponds, & pigs in paddocks, geared to modest-sized humane & free range animal production. This yields multiple traditional products such as wool, leather, dairy & meat as well as providing omega-3, oil fish & other novel biodegradable products.
			• Fermented food introduces more protein/nutrient density (from the microbes) & less energy, but alcohol content needs controlling. Fermented food may decrease levels of (saccharide) prebiotics, for those seeking to control gut distension as in irritable bowel syndrome.
Technology - (cooking, agriculture, mechanisation, research, computer use) [Increased Energy Uptake]	• Cooking softens /gelatinises protein, kills pathogens, improves food utility	• Publically subsidised large ‘high-chemo/tech’, monocultural agri-business farming ruins ecologies, depletes environments & foods plus adds many man-made xenobiotics – all decreasing NRF2 function	• Public Health staff need to work hard with governments to establish programmes of health system & self critiques, & listen to ideas on public health issues from the public, (other) theoreticians, scientists & systems modellers from various disciplines.
	• **Health Food = naturally preserved** from fresh, eg slow cooking, drying, freezing salting, pickling in vinegar/oil, sealing (bottling, tinning) keep many useful nutrients. **Unhealthy Food = processing** by refining, applying excessive mechanical & thermal pressure (& charring, inducing deleterious chemical /Maillard reactions eg advanced glycation end products), extraction with chemicals & inserting anti-nutrient chemicals which damages or poisons, plus eliminates many useful nutrients, in food whilst leaving extremely low ratios of micronutrients: macronutrients.	• Supermarkets & 'fast food' chains tend to be multinational oligopolies, with ‘units on a conveyer belt’ management systems, including inflexible controlling contracts with growers & workers, minimal community involvement	• Public health systems must look at obvious & scientific evidence of environmental/ecological damage, & lack of sustainability of (expansion in) food energy agribusiness, use of pesticides, antibiotics, propping up unstable mono-cultures that will become blighted (kiwifruit, battery chicken, bees), & overuse use of non-sustainable materials that ‘fuel’ climate change as they affect nation’s health
	• Much technology is designed to grow, refine, alter, store, transport & trade in high-energy foods.	• Food marketeers & their ‘high tech’ extensive advertising renders high processed food as the norm	• Public health scientists need to be up-to-date with studies of sustainable, mixed crop, ecological farming using smart methods eg computer technology, large scale composting, & advocate against destructive resource exploitation, such as clearing forest for palm oil & kernel monocultures, which are not required
		• Agribusiness is promoted by those who control economies & is normalised in the media.	• Public health scientists need to know that metabolomics technology can be used to study food & body nutrient status & health. (see healthy food grading, below)
			• Public health systems need to be in contact with those of other nations – as 1/2 world’s food is wasted not counting millions of tonnes of fat energy on human bodies. They should be publishing this fact & coordinating science to combat this
			• Public health should step out of its comfort zone & engage theoreticians to assess new theory & re-analyse data already present, with (mathematical systems) modelling studies
			• Public health physicians, workers & researchers need to scan the evidence closely for health benefits & blow the whistle on /not support or endorse vast public/private funding for research. Such funding is often for high tech ‘industry branding’, that does not clearly have an over-riding, primary health outcome.
			• Simplistic mono-nutrient/supplement/drug product & genotype matching industries are not real science-evidence based & are not contributing to public health science.
Co-Option of Numerous Micronutrients to Modulate the Human NRF2 System (human adapted) [Increase Energy Efficiency]	• Theoretically, the human NRF2 is super energy efficient possibly initially to accommodate the brain’s use of energy. This is due to the modulation by numerous food micronutrients.	• A continuous dietary supply of high micronutrient food with the minimum xenobiotic load is the way of deferring degenerative diseases of all types. The development of ‘technology”, driven by the same cortico-limbic-striatal system, has skewed the food intake too far from the requirements of the NRF2 system. The two systems are no longer complimentary, with the former disrupting the function of the latter.	• Public health staff know there is something seriously wrong in the area of obesity, diabetes, CVD & cancer, that their public health messages are not working, & programmes are not getting management correct with respect to nutrition.
	• What is known is that the abundant use of food micronutrients affords increases a very high level of metabolic protection	• Metabolically, the forager situation is ideal. With good environmental & social conditions & plenty of physical activity, maximum physical & socio-cognitive potential & longevity should be reached.	• Public health always needs theoretical, broadly educated scientists who understand biology to independently assess many disciplines that contribute to population health - including current anthropology, psychology & appropriate (cancer) science.
	• The expanded cortico-limbic-striatal system causes craving of energy dense highly attractive food which leads to neglect of healthy whole food.	• With the above, & normal exposure to the natural environment, allergies, auto-immune & cancer rates should be decreased. Children with asthma & allergies may benefit from having pets.	• Public health scientists have enough evidence to acknowledge that degeneration +/- obesity occurs early with extreme low micronutrient to macronutrient ratio food, as seen in many westernised countries, & to take action with pregnant women, their babies, children & teenage years for the next generation.
	• Oxidative Stress control, hyper-efficient cell maintenance, & repair, allows healthy longevity with minimal tumour development or degenerative change - unusual in modest sized mammal.	• Learning to eat tougher, tasty, highly varied & nutritious food is hijacked by over-palatable, processed, normalised food. Children, youth & their offspring are propelled towards inadequate nutrition & shorter, unhealthy lives.	• Public health scientists will now understand that the signs such as obesity enhanced polycystic ovary syndrome & teenage TIIDM, will occur earlier.
	• Stable Replication - highly functioning immune cells in infection control, & dysplasia prevention.	• A high antioxidant diet allows slow growth means & high cytoprotection over a long life. It sets the scene for menopause. Without lifelong micronutrient rich food any inherent cell reserve is depleted at middle age.	• Public health systems really need to help the poor & ensure that increasing disparities already present between wealthy & poor are decreased – otherwise this is not public health
	• Repair work undertaken by the NRF2 (eg removing oxidised lipoproteins preventing arterial plaque) uses plenty of energy, but is deferred due to lack of micronutrients.	• Lack of micronutrient dense food post-agriculture, with variable energy & variable protein led to starvation, stunting, vitamin deficiency disease & susceptibility to acute/chronic infection	• A related public health issue is increasing neurological disease
			a) psychiatric diseases of attention deficit/ hyperactivity disorder & many learning & conduct (cognitive disorders) have relationships to suboptimal in utero, infant & childhood nutrition
			b) it is known that prevention of MetS in middle age decreases Alzheimer’s disease. Luckily humans have a good repair mechanism in the well supplied NRF2 system. It is never too late to gain some benefit from increasing nutrition quality.
	• Extra unoxidised lipid is stored firstly in subcutaneous safe adipose tissue, then forced into ectopic sites such as liver, locomotor & heart muscle, & other tissue.	• Man-made persistent toxins lead to death, sub-acute illness, chronic disease, CVD, neoplasia or cancer.	• Public health staff will need to understand that oxidative stress along with the supervention of a mal-activated immune system may be behind high rates of *communicable* acute infectious disease. A poorly fed host, chronically exposed to many chemicals, such as diabetic immune compromised individuals is then exposed to contaminated low nutrient, factory farmed meat – that food poisoning is a problem of compromised host & food. Chronic infections disease such as pulmonary tuberculosis is also higher in poorly fed populations
	• The theory promotes the idea that the lack of adequate micronutrients in humans’ diets & excess xenobiotics is the cause of MetS. In recent decades dietary energy overload has occurred & MetS is now coupled with obesity. This malnutritive obesity or 'malnubesity' is epidemic.		
	• On increased consumption of a high micronutrient food diet by individuals who are obese, ectopic & central fat appears to be mobilised in order to contribute to overdue repair work throughout the body.		
Nomad & Forager cultures (human adapted) [Increased metabolic/energy efficiency & Increased uptake]	• NRF2 exposure to wide variety of plant (& animal) foods with co-option of antioxidants & wide exposure to natural toxins & adaptation to managing them.	• Increasing exposure to large varieties of food gradually allowed the co-option of antioxidants & consequent energy efficiency & management of plant toxins.	• World public health policy should learn from nomadic, forager, so-called subsistence & local ‘low tech’, farming societies, & protect from deleterious ‘nutrition transition’ to westernised food patterns
			• Such communities protect regional environmental & food resources for the world, & they need: their land to be protected from resource exploitation, remain little disturbed, to be encouraged to feed themselves whilst maintaining biological diversity & stocks
			• If energy & protein aid is needed for displaced, war or natural disaster exposed peoples, high nutrient local herbs & spice supplementation should be encouraged, or these condiments added to the food aid.
			• Local community gardeners should learn from low tech farmers working in other countries.
Other metabolic management mechanisms (hominoid/human specific) [Energy Efficiency]	• Humans have lost enzymes to synthesize vitamin C (an energy requiring process). Also vitamin C, a sugar based vitamin, yields energy. Vitamin C may inhibit fat production from fructose.	• Vitamin C in fruit (& probably other plant micronutrients, fibre, & other plant food eaten concurrently) inhibits fat deposition from fruit sugars.	• Public Health workers around the world, to their credit, are gradually gaining traction on advice, & in some areas regulation, to decrease sugar content of diets, especially fructose & sucrose in sugary drinks. This is an example of solid basic science evidence being brought into clinical & public health arenas.
	• Uric acid (high levels in hominoid serum) is antioxidant but in high levels, especially in cells, pro-oxidant. Levels markedly increase on fructose & recently found, glucose (& ?starch) ingestion, & rapidly forms liver fat.	• Consumption of high fructose corn syrup, sucrose in sugary drinks, is a major problem for liver, muscle & general fat deposition. Refined starch may be similar.	• Public health is yet to navigate the processed food companies in order to bring the role of food micronutrients, the likely major determinants of health, to the fore. This needs doing as soon as possible. There is, plenty of evidence from Mediterranean diet studies.
	• Vitamin D metabolism is related to prevention of central obesity, diabetes, CVD, infection & cancer.	• Vitamin D appears to be associated with obesity, & relates to glucose regulation, metabolism & immune function (especially skin infections & cancer prevention)	• Public Health messages & programmes need updating & integrating. Obese populations with MetS also usually have low vitamin D levels, especially with darkly pigmented skin, & those who live in crowded, poor housing estates, with few parks & work indoors. As bowel cancer is more common in these groups, & vitamin D can decrease incidence & they may need *more* careful sun exposure. This manoeuvre may have an overall greater benefit than restricting all ethnicities from sun exposure to prevent individuals with lightly pigmented skin from melanoma. People can understand a message on careful, non-burning sun exposure if based on their skin pigment when the issue is of biology not ethnicity. NB Recent Vitamin D & calcium supplements do not seem to be preventing the above, nor fractures, adequately. Graded real skin exposure to summer sun is needed
	• High levels of antioxidants (superoxide dismutase) & fluxes of brain glutamate (metabolism of energy & neurotransmission) are present in humans.	• Rapid & efficient antioxidant activity is especially highly developed in the brain.	• Genetic-epigenetic changes are present in the high glucose consuming brain, however, the brain is able to metabolise ketones during starvation.
**Expensive tissue trade off**			
a)Smaller, simpler gut but still specialised [Energy Conservation].	• The human intestine is a less specialised organ, with no foregut fermentation (no ruminant cellulose digestion), & modest protein fermentation. It is however, very flexible & adaptable	• Gut health itself, let alone the whole body, depends on food micronutrients & fibre (the latter which often carry minerals & vitamins) for normal metabolism & weight	• Public health can emphasise in its public campaigns that eating healthy diets with lots of natural whole food fibre & phytochemicals will keep the symptomatic bowel dysfunction (constipation & irritable bowl) rates in check
	• With a high plant & whole food diet, beneficial *Bacteriodetes* microbes predominate. This is associated with better toxin & pathogen management, & normal weight & metabolic health. Appropriate micronutrients & energy are absorbed, but minimal microbial inflammatory particles. The converse is true with a low quality, energy dense diet, where *Firmicutes* predominate, & metaflammation may arise from plasma microbe particles & DNA.	• Gut motility depends on large groups of micronutrients & fibre. It is under recognised that vegetable and non starch seeds (such as nuts & flax/lin seed) fibre prevents slow bowel transit, constipation & haemorrhoids.	• Public health messages should stop spreading fear of normal environmental microbes/dirt/animal dander/etc ingested with low xenobiotic foods which stimulate normal gut & body immune development. Realistic, plausible, up-to-date science shows these immune stimulators reduce allergic disease eg asthma, & autoimmune diseases of Crohns & rheumatoid type which are already increased in those with MetS.
	• The gut needs appropriate immune stimulation to reduce allergic & autoimmune diseases.	• Immune stimulation occurs by exposure & processing of variably noxious food, microbes, environmental particles & chemicals. Thus the body distinguishes pathogenic items form self. This process is known to be pivotal in preventing allergic & autoimmune diseases.	• Public health messages must start spreading the truth about the hyper-hygiene theory. Antibiotic chemicals & pesticides in daily household & general use are killing off normal microbes. Restriction of normal developmental exposure to natural environmental products, & exposing humans & their microbiota to toxins is probably behind far more disease that mild infection.
		• Normal immune stimulation occurring by exposure & processing of variably noxious food, microbes & natural xenobiotics is healthy. This is disrupted by many man-made persistent chemicals. These & medicinal antibiotics are toxic to our commensal microbes, & our NRF2 metabolism, & should be avoided.	• Not attending to this truth feeds into the profits of highly advertised ‘sanitation’ products, including mouth washes. Sensible advice is to wash/wipe items with water or minimal mild environmentally friendly soaps/detergents until they look & small clean
		• Weight gain in malnourished children given antibiotics may occur but if linear growth is not, the increased weight gain may be metabolic risky central fat.	• The only time very careful microbe killing should arise is where there are truly immunosuppressed persons or sites where surgery takes place ie hospitals.
b)“Over-fat & Under- muscled”; Humans are Fat From Birth (human specific) [Energy conservation]	• Muscle is a high metabolic rate tissue & generates work & heat whereas subcutaneous fat insulates body & efficiently stores, rather than uses, large amounts of energy.	• Metabolically safe lower body/peripheral subcutaneous fat depots develop - especially in pregnant/lactating women with 2 brains to buffer.	• Public health staff should understand & lead the community/primary health care professionals’ understanding of the different fat store morphology, health & health care implications
	• This is probably an expensive tissue trade-off – “muscle for fat to conserve energy for the brain”.	• There are gender, age & ethnic group differences, eg buttock fat in some black groups, hip & thigh in white groups – mostly women.	• Treatment needs for the peripherally over weight are largely about locomotion, musculoskeletal, hygiene & body shape acceptance problems. Conversely the centrally overweight need urgent nutritional coaching & enablement to increase whole food intake for improved weight loss & metabolic health
	• Human neonates are born fat – adipose tissue fat is a probable energy buffer for the brain which uses > 85% of the body’s energy at birth.	• These depots can be very large, & sometimes involves total body subcutaneous +/- central adipose (the classic Pickwick shape)	• Some individuals/groups having high subcutaneous fat reserves (gatherer phenotype), need low impact, upper body physical activity, (walking and carrying) whereas the slim-limbed, centrally obese (hunter phenotype) should work up to more intense, locomotor & upper body based activities, (hunter phenotype) which also tends to decrease visceral fat
	• By storing fat safely away from high metabolic tissue (brain, liver, muscle, pancreas) the micronutrient to macronutrient ratio becomes larger & healthier.		
Slow Growth and development [Energy Conservation]	• Human infants grow & develop slowly, possibly to allow adequate continuous supplies of energy to be channelled to the brain	• Slow growth allows time to accumulate fat reserves & in turn ensure the brain always has enough energy to develop. Note human breast milk is geared towards ‘brain nutrients’. NB partial weaning is earlier (4 rather than 6 months) in humans due to high energy demand of the infants brain	• Public health science needs to be geared to understand that humans grow & develop slowly, but have good powers of repair – & nutrition is behind this.
	• A co-adaptation, to the ability to live long, is menopause, which prevents long lived women from breeding past 50y, possibly enhancing care of grandchildren.	• Slow growth & development has allowed the transmission of knowledge & culture	• Public health services need to increase aid to establish breast feeding in all women but particularly the obese with MetS, for whom this is more difficult. Large breasts & less healthy pregnancies & infants make this help all the more important – breast milk helps decrease the risk of the next generation being obese. Note - milk rarely ‘dries up’ unless suckling (or breast milk expressing) stops.
		• Slow growth & delayed reproduction can only occur if there are very robust cell protection & maintenance mechanisms vis à vis the micronutrient dependant NRF2 cell protection system.	• Public health workers may need to promote *total* breast feeding of babies to 4-6 months then *partial* breast feeding for longer for 18 months+, ideally.
		• Healthy longevity depends on a high ratio of micronutrient to macronutrients - degeneration sets in after youth when inherent reserve in cells declines	• Feeding children at (pre-) school is likely to be very cost effective. Improved nutrition whilst growing & developing is known to return children closer to their physical , metabolic & cognitive potential.
		• When the micronutrient to macronutrient ratio is extremely low from preconception, central obesity & lean tissue deficiency rapidly associate with degenerative change; even pre-maturation. Although puberty is early with MetS & TIIDM , polycystic ovary syndrome & male gonadal hypo-function impair fertility. Teen pregnancies are decreasing in some groups with low socioeconomic status, poor nutrition & obesity. At the same time poverty is over represented in various ethnic groups, so falling fertility and not reaching their reproductive potential may be an ethnic survival problem.	• Public health should have policies that makes the most of the NRF2 potential for regeneration & repair - this means that it is worth feeding those with likely substandard nutrition eg prisoners, other wards of state, hospital & rest home residents healthy food – for social, developmental & ultimately economic reasons.
			• Public health should ensure that the whole population is feed well from pre-conception to old age as it will save money by having a population at lower risk of physical, neurological & mental health dysfunction, & will keep people in work for longer.
			• Public health study, action & advocacy on nutrition, from food production planning to the meal table, will help keep far more of the human race disease free, reproducing successfully & help ensure good quality survival.

### A negative projection

Without understanding the likely cause of MetS, the future prospect will be to continue on the current trajectory. One American public health publication warned of the current scenario of a degenerative disease epidemic 60 years ago [70]. Furthermore, a report was recently published on the ‘Shorter Lives, Poorer Health’ in the USA. The USA compared poorly with 17 other wealthy countries over the last 30 years, in spite of massive health spending. The report underlines nine areas of attention, five areas of which are: adverse birth outcomes, obesity/diabetes, CVD, lung disease and disability, and possibly two more; drug-abuse mortality and HIV/AIDS, which may relate to the composite unifying theory [71]. We know the ‘developing countries’ have escalating food and general pollution problems [55].

Increases in refined energy food and industrial toxin production will cause further ecological degradation, deforestation, desertification, pollution and acidification of the waterways including global climate change, indicating that MetS is an environmental problem [72,73].

In poor countries or communities with few industrial regulations, high levels of environmental and occupational toxic chemical exposure [55,74,75] are telescoped in time and coincide with addictive, extremely micronutrient depleted westernised refined energy diets, as well as other poverty stresses. This combination of general westernisation and nutrition transition escalates MetS, CVD and cancer rates [62,75].

Most humans, particularly in less wealthy populations, are now dependant on food products derived from chemically supported and engineered agribusiness monocultures, often grown in low socioeconomic countries [55]. These are at increasing risk of catastrophic failure, often as weakened crops or stock predictably fall prey to rogue microbes [76]. Global climate change is another area of environmental damage, and should be factored into public health action [77].

Individuals will increasingly be exposed to maternal malnubesity and circulating pollutants in utero [54]. They are at risk of suffering cognitive deficits and behavioural dysfunction [78], early MetS (TIIDM in teens), disturbance with reproduction (polycystic ovary syndrome and male hypogonadism) [79], and early degenerative CVD and cancer [78].

### A positive projection

Good science on nutrition, the environment and sociology, for example, is available to the public. Civil society groups are already collating ground swells of public opinion via the internet and other communication channels. These can influence governments to reduce corruption [80], warfare (death, injury, starvation, destruction, pollution) and unfair public or government subsidies to large agribusiness [81] and related industries (Table 1). Transparent regulation is required to make the profiteering refined energy food conglomerates provide minimum standards of nutrition, and be progressively taxed on products that do not meet these requirements [62].

Perhaps the most important area is for public health money to be diverted to work with all pregnant women, and help feed poverty stricken mothers and children healthy, non toxic food [71].

Preventing some of the world’s already grown and harvested food, of which half is estimated to go to waste [82], would solve the problem of converting further natural ecologies into unsustainable resource ‘mines’.

It is imperative to stop the rapid nutrition transition to westernised food of cultures which are still farming and foraging traditional whole foods, and protect their land and way of life. This also serves to protect food patterns and environments from westernising exploitation, often destruction, and conserves world resources and knowledge. In fact, for the western or European populations, which have taken generations to industrialise their food, it is time for them to quickly reverse their own nutrition transition back to traditional and forager whole foods.

There is plenty of evidence for scientifically smart, technologically assisted, sustainable mixed organic, ecological farming [69], and even sustainable industry [83]. Many of the traditional food - farming and preparation – methods [84] may be able to be reclaimed and reinstituted, especially by low income countries. Increased types and numbers of programmes to construct more new, simple but smart, environmental already-designed-technologies can be introduced to these areas. Labourers on such farms producing food for the local community should be much healthier than industrial factory workers making components from toxic chemical for arguably unnecessary items.

Major media will be exposed as being owned by large industrial magnates [85] where reporting on much science, medicine and technology is designed to encourage consumption and investment, rather than health. If local and national media can be freed from influence of the wealthy conglomerates [85], then non-environmental agribusiness will no longer be framed as the more positive pathway [86]. Education and promotion of more ecological methods of food production that are likely to lead to better farming, healthy food and less obesity [87]. Media framing of the ordinary, low income family, where obesity is common, would no longer be pejorative [88,89] (Table 1).

Obesity management and prevention must target the most at risk groups – who are impoverished, deprived of all resources, including good education, often treated unjustly, and often of non-dominant cultures. World efforts need to be increased to regulate corrupt human, environmental and resource exploitation by large multinational corporations, as a first step [90]. This would have an effect on resource wars. Serious new global health modelling on new ecological ways of running economies needs considering. Communities can regroup if left alone. Plans on managing obesity and inequity are appearing [91].

Government public health initiatives can work towards making it easier for individuals in communities by regulating local food producing, processing and marketing industries. Another initiative would be to show the community the value of whole food to health by subsidising fruit and vegetables at least to the levels of other consumables [92]. City councils can provide spare land for community gardens (Table 1).

Healthy, inexpensive whole foods can largely replace refined energy food. This, together with minimising the exposure to persistent pollutants, significantly alters the micro to macronutrient balance and normalises NRF2 metabolism. These environmental and behavioural life status (rather than lifestyle) changes can effect a surprising amount of metabolic repair [93] at any age or stage of disease. Centrally obese individuals with minimal end organ damage can reverse, or bring into remission, TIIDM.

Some specialist addiction management principles and practices will need to be used for the already overweight, possibly with medical and pharmaceutical interventions [94].

Addiction counselling techniques should be used in most cases. Long term assistance in a low stress, non-judgemental, therapeutic environments should be provided [95]. Once individuals have been overweight or obese for some time, and are distressed, most are relieved to classify the problem as addiction. They easily understand that the current environment is pushing most people to overeat ‘junk food’ and that the metabolic problems are due to a lack of food micronutrients. This is especially so when reasons for the types of foods that engender addiction, and other components of addiction, are explained [94,96] – and often patients recognise them. The next step is explaining that the brain pathways that lead to addiction do not link with logic - ‘addiction can’t be reasoned with’. Thoughts around guilt are usually misplaced and unhelpful, but are encouraged by external influences such as media [89]. Experimenting with new reality testing behaviours, especially with regards to self-worth, can be helpful. Working with patients on advance planning to set rules for abstinence and replacement with non-addictive but healthy, medium energy, pleasant food can work. Practice and repetition over time to establish new patterns or habits is important. However, as most patients know, stress can lead back to ‘comfort eating’ – a euphemism for addiction [97]. Withdrawal symptoms and relapse can often be predicted and managed.

Anti-addictive medication is likely to be helpful for many who are already overweight. Phentermine, an old but understudied medication [94,98,99], partially and selectively dampens appetite for rich foods, which along with naloxone [100] and lorcaserin [101], have adrenergic, antiopiate, serotonergic and ultimately dopaminergic effects [102]. The fright, fight or flight response appears to be activated, which tends to decrease saliva flow, and appetite possibly selectively for refined energy food (Table 1).

Metabolic medication has been shown to augment basic energy pathways. Metformin has ‘antioxidant’ [103] type effects, which decreases insulin resistance [103]. It is associated with modest weight loss and confers a significant degree of improvement in MetS and prevention of CVD and possibly cancer [30]. Allopurinol decreases the pro-oxidant enzyme xanthine oxidase [104], directly or indirectly via the NRF2 system. Medications to primarily ‘normalise’ blood pressure, glucose and lipids, MetS markers, can only be temporary measures. When hyperinsulinaemia is already present, the hypoglycaemics, high dose insulin and insulin secreteagogues, sulphonylureas, can have the adverse effects, worsening other aspects of MetS, such as central obesity.

Once individuals are on a whole food diet, daily physical activity will cause normal muscle microdamage and minor arterial endothelial shear disruption-associated oxidative stress [105]. This slight stress will stimulate repair (hormesis [32] again), effectively signalling total body upkeep [29].

Educated primary care health professionals should be leading general and specific patient support, coaching and monitoring of healthy food and physical activity plans.

### Summary

In summary, it is proposed that as the human brain became enlarged, which increased its energy demands on the body, a human specific ‘dual system’ and other co-adaptations were required to provide extra energy for the brain.

To increase energy uptake, the cortico-limbic-striatal system, an expanded neural network, has driven humans to devise wide-ranging technologies to make available extremely refined energy food for the brain. The same system is probably involved in addiction, initially to refined energy food, but also in compulsions to seek and maintain power over resources (and other humans).

Ultimately, food technologies have processed food until it is unregognisable, largely eliminating micronutrients, leaving refined energy dense foods. However, humans depend on micronutrient dense food for their health and longevity. The NRF2 system’s power to maintain an extremely high level of antioxidant cell protection and (partially) detoxify persistent man-made chemicals depends on relative and absolute food micronutrient sufficiency.

There has been a progression of modest micro to macronutrient ratio decline, with the transitions to agrarian based cultures over the ages, leading to stunting. This was aggravated by work and residence in crowded, sunless habitations, with poor access to fresh fruit and vegetables. In towns, on voyages, and during and after migration, especially with insufficiencies of in vitamin intake, infectious plagues became frequent.

In the industrial age the above continued, but became combined with additional large, uncontrolled releases of toxic man-made environmental pollutants. These initially caused acute toxicity, with significant morbidity and mortality. Over time, pollutants released were less acutely toxic but more numerous, with subacute oxidative stress and metaflammation occurring. The largest organ of the body, the endothelium, especially, was damaged, with atherosclerotic plaques accreting on the large, shear stressed arteries of the aorta, heart and brain, and lower limbs causing CVD; IHD, stroke and peripheral vascular disease.

Early in the 20^th^ century CVD incidence and prevalence increased rapidly, although it is still a leading cause of illness, disability and death in the 2010’s. Over longer lead times still, from the mid-20^th^ century on, cancers developed from accumulated DNA damage associated, at best estimates, with constant low grade epithelial exposure to xenobiotics, and low food micronutrient intake. Cancer incidence increased, with increasing prevalence but less mortality, possibly due to palliative, and some curative, care.

In the later 20^th^ to the early 21^st^ century arose populations who made an extremely rapid nutrition transition to westernised food with extreme micronutrient to macronutrient imbalance, and chronic exposure to myriads of persistent man-made environmental pollutants; they tend to develop extreme central obesity-related MetS. CVD rates and cancers are increasing again in proportion to central obesity-related MetS.

## Conclusion

The composite unifying theory includes the ‘dual system’ theory and other co-adaptations, as an explanation for malnubesity, a condition of excess fat accumulation but with concomitant insufficiency of vitamins, minerals and plant and other micronutrients. An understanding of the composite unifying theory can be used as a basis to remediate the current MetS epidemic.

Public health and primary health care professionals can help with refined energy food addiction, utilising current addiction management techniques modified from alcohol and drug programmes, as well as and general education and support for generally unlimited whole food intake, without calorie counting. Non-judgemental coaching for healthy eating together with adequate physical activity will improve health to a large degree (Table 1).

Government public health and environmental defence departments need strengthening and protection from private company lobbying to reduce or influence their charters (Table 1).

Enabling more families to grow and prepare healthy whole food in community gardens or ‘buying local’ from environmentally sound and sustainable, ‘smart’ farmed food, which has minimal synthetic chemicals added, will also be important.

Town planning including public transport engineering and architecture regulations can be improved for more environmentally appropriate structures, accessible parks and active transport systems.

It could be hoped that a number of public health, primary care, human nutrition, evolutionary medicine specialists, and traditional farmers and environmentalists will be interested in the current discussion, and not surprised. Many have known that MetS management, in all areas, is inadequate. Ideally health, environmental and wider economic cost benefit modelling will convince some powerful countries’ governments that regulating and reigning in the global exploitative industries, to make way for sustainable industries and a healthy food culture, will be well worth their while.

## Competing interests

The author declares that she has no competing interests.
